# Fully Automated Bone Age Assessment on Large-Scale Hand X-Ray Dataset

**DOI:** 10.1155/2020/8460493

**Published:** 2020-03-03

**Authors:** Xiaoying Pan, Yizhe Zhao, Hao Chen, De Wei, Chen Zhao, Zhi Wei

**Affiliations:** ^1^School of Computer Science and Technology, Xi'an University of Posts and Telecommunications, Xi'an, Shaanxi 710121, China; ^2^School of Computing Sciences and Computer Engineering, The University of Southern Mississippi, Hattiesburg, Mississippi 39406, USA; ^3^Department of Computer Science, New Jersey Institute of Technology, Newark, NJ 07102, USA

## Abstract

Bone age assessment (BAA) is an essential topic in the clinical practice of evaluating the biological maturity of children. Because the manual method is time-consuming and prone to observer variability, it is attractive to develop computer-aided and automated methods for BAA. In this paper, we present a fully automatic BAA method. To eliminate noise in a raw X-ray image, we start with using U-Net to precisely segment hand mask image from a raw X-ray image. Even though U-Net can perform the segmentation with high precision, it needs a bigger annotated dataset. To alleviate the annotation burden, we propose to use deep active learning (AL) to select unlabeled data samples with sufficient information intentionally. These samples are given to Oracle for annotation. After that, they are then used for subsequential training. In the beginning, only 300 data are manually annotated and then the improved U-Net within the AL framework can robustly segment all the 12611 images in RSNA dataset. The AL segmentation model achieved a Dice score at 0.95 in the annotated testing set. To optimize the learning process, we employ six off-the-shell deep Convolutional Neural Networks (CNNs) with pretrained weights on ImageNet. We use them to extract features of preprocessed hand images with a transfer learning technique. In the end, a variety of ensemble regression algorithms are applied to perform BAA. Besides, we choose a specific CNN to extract features and explain why we select that CNN. Experimental results show that the proposed approach achieved discrepancy between manual and predicted bone age of about 6.96 and 7.35 months for male and female cohorts, respectively, on the RSNA dataset. These accuracies are comparable to state-of-the-art performance.

## 1. Introduction

Bone age assessment (BAA) may provide important clinical information for skeletal maturation estimation, especially for the diagnosis of endocrinological problems and growth disorders [1]. The discrepancy between bone age and chronological age indicates the abnormalities in skeletal development. In clinical practice, radiologists perform BAA through examining a left-hand-wrist X-ray image. Historically, two BAA methods, Greulich and Pyle (GP) method [2] and Tanner Whitehouse method (TW2) [3], are widely used in clinical practice. The GP method compares the patient's X-ray image with a representative age atlas and determines the bone age. In the TW2 method, twenty regions of interest (ROIs) located in the main hand bones are taken into consideration for the bone age evaluation. All these procedures are tedious, time-consuming, and prone to observer variability.

**Figure 1:**
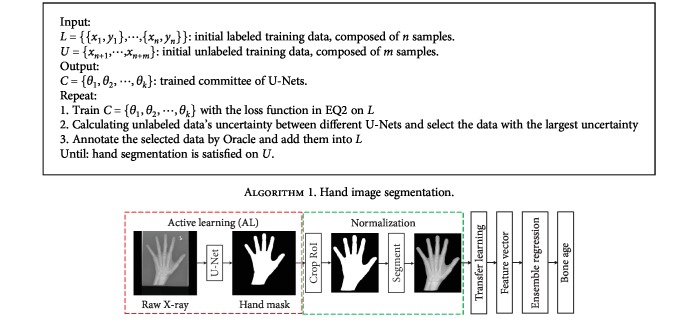
Overview of the proposed automated BAA deep learning pipeline.

As we know, deep learning has been applied to computer vision task and achieved drastic performance improvement. In this paper, we propose a method which learns real latent features of hand X-ray images and facilitates the feature capture to perform BAA. At the beginning of the method, we train the U-Net neural networks to precisely segment hand image from radiographs and eliminate insignificant information in raw X-ray images with an active learning technique. Then, we use pretrained deep Convolutional Neural Networks (CNNs) to extract high-level features with a transfer learning technique. After that, ensemble learning is employed to perform BAA with different base regressors. Finally, we evaluate the overall performance of our approach across different models. The proposed method pipeline is shown in Figure 1. The experimental results demonstrate that the proposed method is more robust and achieves a state-of-the-art performance.

## 2. Review of BAA

The conventional BAA approaches could be categorized into GP and TW methods. Some traditional machine learning methods have been applied to a BAA approach, such as support vector machine (SVM) [4], SVM with cross-correlation [5], and support vector regression [6]. Besides, the most prevalent and widely used software for automatic BAA in Europe is BoneXpert [7]. This system works based on a shape-driven active appearance and TW RUS-based approach. However, it is sensitive to image quality and does not utilize the whole set of hand bones, although all of them are important for skeletal maturity assessment. In summary, all of the aforementioned works demonstrate a lack of accuracy.

Recently, motivated by the success of deep Convolutional Neural Networks (DCNN) in image classification, studies in medical imaging have been exploring such methods. Rucci et al. uses an attention focuser and a bone classifier in a neural network to extract features of carpal bones and performs BAA [8]. While the method presents the neural network as a useful technique for classification in the TW2 method, the approach does not achieve satisfying results with an error rate of 1.4 years. In 2012, Mansourvar et al. developed a fully automated BAA system that uses compression techniques base on histogram methods [9]. This approach works on an image repository to perform similarity measures and uses a content-based image retrieval method for image processing. Because the proposed method does not perform precise hand segmentation, it is not reliable for images with poor quality or abnormal bone structure. Deep learning-based methods allow avoiding feature engineering by automatically learning the hierarchy of discriminative features directly from a set training data. DCNN has been successfully applied in the bone age estimation [10–12]. All of above methods are end-to-end learning architecture to estimate bone age using DCNN.

Despite some methods yield very accurate results, most existing methods suffer from two main limitations:

1. Most of the above methods operate on coarse segmentation of hand images, which might mislead BAA toward focusing on irrelevant ROIs
2. Most of the proposed approaches use hand-crafted features, such as HOG feature, LBP feature, and Harr feature, thus constraining regressor or classifier to use low-level X-ray image features rather than the higher and deeper latent features. This semantic gap always limits the generalization capabilities of BAA systems

## 3. Active Learning for Hand Image Segmentation

To perform BAA, we first extract a precise region of interest (ROI), a hand mask, from the raw X-ray image. We then remove all irrelevant objects which may mislead model training. It is necessary to establish a nonlinear mapping from original X-ray images to hand ROIs for eliminating noise in raw X-ray images. Recently, deep learning solutions have been successfully used in a multitude of medical image semantic segmentation tasks [13]. Even though significant performance improvement has been achieved, a bigger annotated dataset is essential for model training. However, in practice, the task of annotating medical images is tedious, time-consuming, and may need expert knowledge. To alleviate the annotation burden, we employ a technique called active learning (AL) to actively select unlabeled hand radiograph during the training procedure and ask for human annotation. The proposed method achieves high performance on hand segmentation with as fewer annotations as possible.

### 3.1. Active Learning with Query by Committee

**Figure 2:**
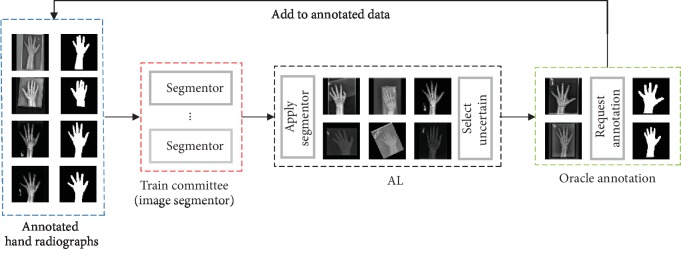
Overview of proposed deep AL framework for hand radiograph segmentation.

In this paper, we propose a new framework for hand radiograph segmentation using the AL strategy with a limited amount of labeled training data. The flowchart of the proposed framework is shown in Figure 2. Let labeled dataset, *L* = {{*x*_1_, *y*_1_}, ⋯, {*x*_*n*_, *y*_*n*_}}, be a collection of hand radiographs, {*x*_*i*_}, and corresponding labeled hand binary masks {*y*_*i*_} (background and hand mask are represented by 0 and 1, respectively). We define *U* = {*x*_*n*+1_, ⋯, *x*_*n*+*m*_}, be a collection of unlabeled data. The selection of samples in *U* is determined by the AL framework. During the training iteration, the most informative samples which are beneficial for model training are selected preferentially. Our task is to train a nonlinear mapping function, a deep neural network, with *L* and make use of *U* to refine the parameters in the network.

The main hypothesis in AL framework is that active learning can judge which data contain the most abundant information for Oracle annotation. The process of AL is like a pupil learns a curriculum. Along the learning process, the pupil spontaneously determines which sample is hard and then asks teachers which sample is well studied. In this setting, AL does not require human to annotate all training data, but only the most uncertain data in the training process.

In practice, query by committee (QBC) is a common strategy in the field of AL [14]. The fundamental prerequisite behind QBC is to minimize the version space [15]. QBC contains a committee *C* = {*θ*_1_, *θ*_2_, ⋯, *θ*_*k*_} of network models, all of which are trained with the same labeled dataset *L*. In BAA problem, we demonstrate *θ*_*i*_ as a model which learns nonlinear mapping from hand radiograph to hand binary mask. We define each model as a deep neural network. In QBC framework, every member cooperates to determine which unlabeled data need to be annotated by Oracle after each training epoch. Our goal is to train them and develop sound cooperative relations. After each training epoch, all members in *C* jointly determine the uncertainty of each unlabeled datum.

Now, we define an uncertainty measure for the level of disagreement of a committee. Since we train a set of members in the committee, each member can extract features of the unlabeled data. We use a different random seed to generate the initial model parameters for a different member so that in each training iteration, the features extracted by each member are different. In practice, we flatten the feature to a vector, and the feature similarity of two members are as follows:

(1) $$\begin{array}{c} {\text{cosine}}_{\text{sim}}=\frac{\text{vecto}{\text{r}}_{1}*\text{vecto}{\text{r}}_{2}}{\left|\text{vecto}{\text{r}}_{1}\right|\left|\text{vecto}{\text{r}}_{2}\right|}, \end{array}$$

where the vector_*i*_ stands for features extracted by *θ*_*i*_ in committee *C*.

The unlabeled datum with the lowest feature similarity indicates the datum has the most significant information which is supposed to be helpful for the model training. Therefore, the ground truth of such unlabeled datum should be annotated by Oracle and then added into the labeled dataset for the following training epochs.

### 3.2. U-Net for Hand Segmentation

**Figure 3:**
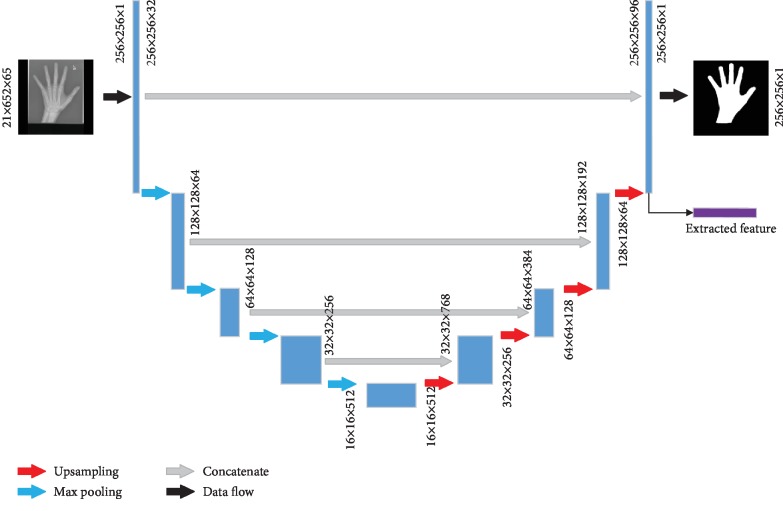
The overview of hand segmentation AL model.

As demonstrated in [13], U-Net is robust in medical image segmentation and needs a smaller number of labeled data. U-Net uses convolution layer to automatically extracted features and uses skip connection to remove the cropping operation and maintains the low-level image outline information in high-level feature maps [16]. We deepen the network and widen the receptive field to refine the structure of U-Net by increasing the number of filters in each convolutional and upsampling layer. The refined U-Net structure is shown in Figure 3.

In Figure 3, each blue block corresponds to a multichannel feature map. The arrows with different colors suggest different data flow operations. The purple block (vector) represents the deep feature vector of the input image inferred by each member. The vector is used in QBC to calculate the similarity between each member in *C* and to determine whether the input image needs to be annotated or not.

In addition, we optimize the loss function in the process of training a member, i.e., a U-Net. In the field of image segmentation, a pixel-wise loss function is usually used to penalize the distance between the ground truth and the predicted probability map. We define the pixel-wise loss function with a cross entropy formulation:

(2) $$\begin{array}{c} L_{\text{pixel}‐\text{wise}}=\underset{i}{\sum }-y_{i}\log\left({\hat{y}}_{i}\right)-\left(1-y_{i}\right)\log\left(1-{\hat{y}}_{i}\right), \end{array}$$

where  *y*_*i*_ and $\;{\hat{y}}_{i}\;$ stand for the ground truth and the predicted probability map of pixel *i*, respectively. This loss examines each pixel individually, and this helps in speeding up the training for neural networks in comparison to the quadratic loss.

### 3.3. Algorithm Description

In this section, we summarize the algorithm for hand image segmentation.

## 4. Bone Age Assessment Model

Even though CNNs are more commonly used in image classification tasks, BAA is a regression task in fact. Indeed, the essential of CNN is to extract different level features with various convolutional filters. The extracted features are always fed into a softmax classifier followed by several Fully Connected (FC) layers to classify input images. Inspired by the classification task model, we aim to use deep CNNs and traditional regression algorithms to perform BAA.

### 4.1. Hand Bone Features Extracting

The key point in BAA is to extract distinct features from preprocessed hand images. Usually, a large training dataset is necessary to fine-tune a deep CNN. However, RSNA dataset only provides 12611 images, which is a pittance amount of data compared with ImageNet dataset, which contains nearly 15 million images. Consequently, training a high-level feature extractor is difficult on RSNA BAA dataset.

In this situation, we use a transfer learning technique and a variety of models with pretrained weights to acquire features. Transfer learning has been applied to datasets which are similar to large-scale ImageNet dataset such as [17]. Although medical data are different from natural image, transfer learning can be a possible solution for medical data feature extraction. It uses weights trained on images in other domains and infers medical image high-level features through the network pipeline. Recent researches in [18] demonstrate that it is possible to transfer domain-specific knowledge from natural images to medical images and achieve brilliant performance.

Since CNN has been proposed, researchers have designed various deep CNN models. Several state-of-the-art examples are VGG-16 [19], VGG-19 [19], ResNet-50 [20], Inception-V3 [21], Inception-ResNet-V2 [22], and Xception [23]. In this paper, we use the same preprocessing methods in the above networks to preprocess segmented hand bone images. We initialize the six above networks with pretrained weights on ImageNet then use them to extract features of hand radiographs.

By using transfer learning, the high-level feature maps or high-level 3-dimensional tensor of hand radiographs can be acquired from the last CNN layer of the CNN network. We use Global Average Pooling (GAP) to flatten the feature maps into a 1-dimensional vector, and the vector denotes the high-level feature of the images.

**Figure 4:**
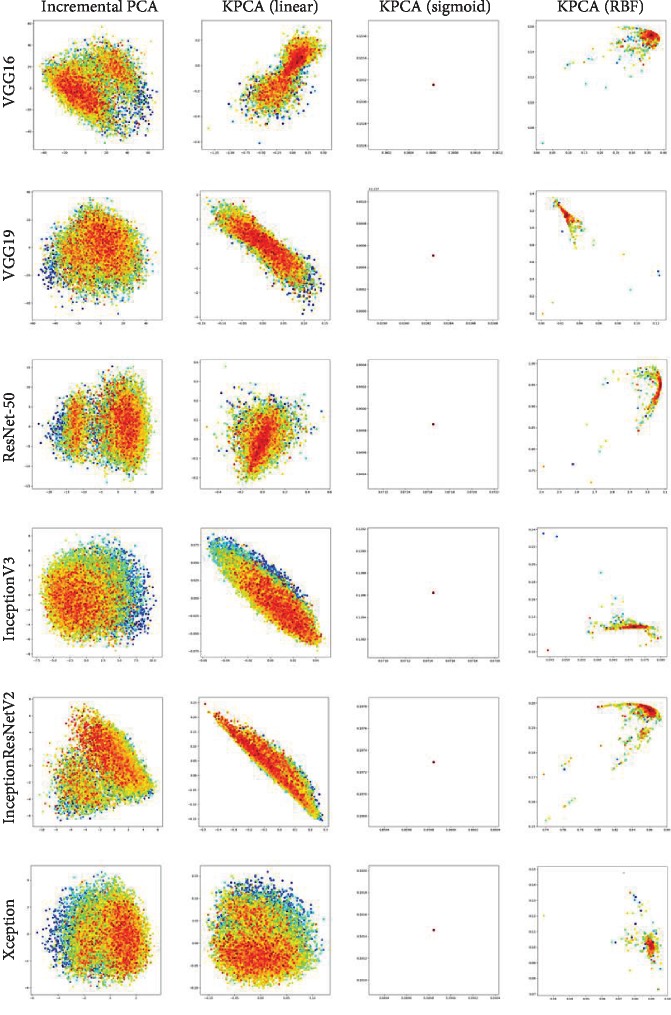
Visualizing features from different models and different decomposing methods.

After extracting the features of hand radiographs, we decompose them into 2-dimensional features by incremental PCA [24] and kernel PCA [25] with different kernel functions. Then, we visualize the 2-dimensional feature distribution. The visualization results are depicted in Figure 4.

In Figure 4, each row represents the features extracted from a specific model, and each column represents scatter plots processed by different PCA methods. Colors in different points represent the data with varying ages of bone. The horizational and vertical axes represent the decomposed feature value. The numbers of features extracted by the last layer of VGG, ResNet-50, Xception, Inception-V3, and Inception-ResNet-V2 are 4096, 1000, 2048, 2048, and 2048, receptively. By applying the PCA algorithm, we decompose the feature dimension to 2 so that we could visualize it in 2D plots. Hence, the meaning of Figure 4 is just to demonstrate which model is suitable for performing BAA, rather than calculates or visualizes the value of extracted features.

From the first column in Figure 4, we conclude that features extracted by Inception-V3, Inception-ResNet-V2, and Xception are easy to distinguish since that data with the same bone ages incline to aggregate into a cluster. In other words, features of similar labels are gathered and ordered. On their counterpart for VGG and ResNet, the data represents with the color of red are prone to gather in two different clusters. This is because all of Inception-V3, Inception-ResNet-V2, and Xception are multiscale deep CNNs. They have a powerful ability to process different size of hand bone in preprocessed hand radiographs and generate distinct features.

A further conclusion is that linear kernel function may be better for differentiating data compared to RBF kernel function.

### 4.2. Bone Age Assessment

With the analysis in Section 4.1, we adopt support vector regression (SVR) [26] and Kernel Ridge Regression (KRR) [27] with a linear kernel function. The penalty parameter of SVR is 1.0, the kernel function of SVR is linear kernel, and the tolerance for stopping criterion is 1*e*-3. The kernel function of KRR is linear, the coefficient of KRR is 3 which leads to a cubic linear function, and the improvement parameter of KRR is 1.0. Before doing the regression task, we scale bone ages from [0, 228] months to a uniform float value [0, 1]. At the inference stage, we project their bone age back to the original range, i.e., 0 to 228 months. Cross-validation is employed at the training stage to prevent overfitting and achieve better generalization performance. We set cross fold as 5.

## 5. Experiment and Discussion

### 5.1. Data Overview

**Figure 5:**
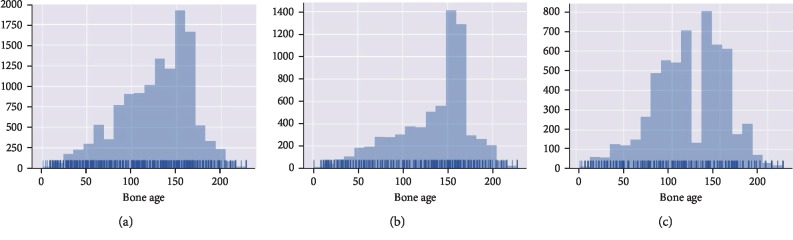
Bone age distribution of (a) full dataset, (b) male, and (c) female. The unit of the horizontal axis is the month.

We obtain hand bone radiograph from the 2017 Pediatric Bone Age Challenge organized by the Radiological Society of North America (RNSA) [28]. The provided dataset contains 12611 left-hand X-ray images with corresponding bone age ranging from 0 to 228 months. The bone age distribution for radiographs of all the dataset, female, and male is depicted, respectively, in Figures 5(a)–5(c). The horizational axis represents the bone age of months which the vertical axis indicates the histogram value of relevant patients.

**Figure 6:**
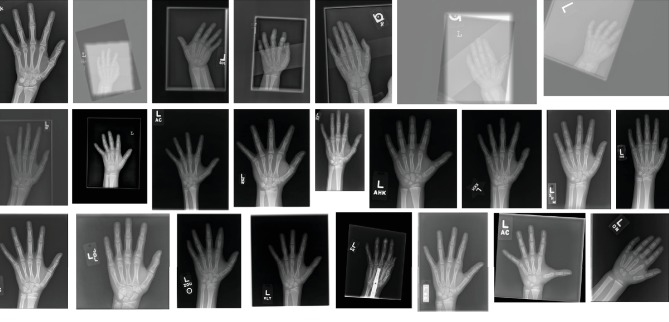
A close-up of part of data in RSNA dataset. Different radiographs vary in size and height-width ratio.

The X-ray data provided by RSNA vary considerably in intensity, contrast, and brightness. A part of the dataset randomly selected is shown in Figure 6. This variance increases the difficulty of training a robust and precise segment for hand image. Furthermore, it prevents algorithms from learning unified and salient features from the radiographs. The optimization of parameters always traps in bad local minima to yield incorrect bone age prediction. In this circumstance, a robust preprocessing engine plays a vital role in data preprocessing, and the standardized images are essential for model accuracy. Performing BAA using the whole RSNA dataset is a challenging task. That is why all the previous works use only selected part of the dataset.

### 5.2. Hand Segmentation

Taking our hardware computation ability and memory space into consideration, we set the committee size as *k* = [3, 5, 7]. That is to say, we train 3 or 5 or 7 U-Nets with the same architecture and initialize their model parameters with different random seeds. At the initial stage, we randomly select 100 hand radiographs from RSNA dataset and manually annotate the hand masks. The training procedure starts with the labeled 100 paired data. Before feeding the data into U-Net, we normalize each pixel by using img = (img–mean(img))/std(img), where *mean* and *std* indicate the mean of pixels and standard variance of pixels, respectively. After each training iteration, we evaluate the similarity of all the unlabeled data between two U-Nets and select the data with the lowest similarity. Finally, the designed interactive training program will tell us which data need to be annotated.

**Figure 7:**
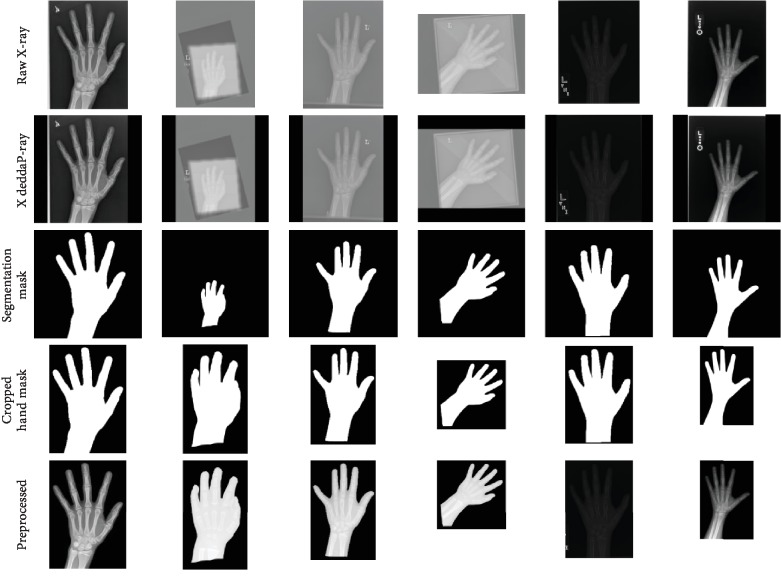
Examples at each stage of preprocessing in the segmentation pipeline.

In practice, in addition to the initial 100 annotated hand radiographs, we annotated another 200 images within the first 20 training epochs. After every training epoch, we annotated 10 radiographs and added them to the training dataset. Then, we trained the committee with another 80 epochs. The value of loss function convergence at a satisfying stage and we visually inspect all the predicted masks and keep all of them. The segmentation results are shown in Figure 7. As demonstrated in Section 2, RSNA hand radiographs vary considerably in intensity, contrast, and brightness. To enhance model performance, we normalize the different grayscale bases by using Contrast Limited Adaptive Histogram Equalization (CLAHE) [29]. Besides, we evaluated the Dice score, sensitivity, and specificity of the segmentation results, shown in Table 1. As a comparison, we use Fully Supervised Learning (FSL) to train the hand segmentation network.

From Table 1, we found that our proposed AL framework outperforms the FSL model with the same number of annotated training data. A further investigation is that the model performance is improved with the increment of the number of members in the committee. The reason behind is that when we use more members to train the model, the difference of learned features between different members is increased so that the probability of choosing the most informative samples is extremely enhanced in the proposed AL framework.

From Figure 7, we observe that the trained U-Net is robust so that it can translate raw X-ray images with different sizes, different contrast, and different brightness to hand masks. Fascinatingly more, we achieve this performance by annotating just 300 images while the RSNA dataset contains 12611 data. In other words, we only labeled about 2.3% data and our improved U-Net with deep AL framework can robustly segment hand masks in various hand radiographs. It is necessary to normalize raw prediction by cropping hand ROIs from segmented images for enhancing model performance. The final preprocessed prediction and raw image with hand mask are depicted in the last two rows in Figure 7.

### 5.3. Bone Age Assessment

With the analysis in Section 4.1, we adopt support vector regression (SVR) [26] and Kernel Ridge Regression (KRR) [27] with a linear kernel function. The penalty parameter of SVR is 1.0, the kernel function of SVR is linear kernel, and the tolerance for stopping criterion is 1*e*-3. The kernel function of KRR is linear, the coefficient of KRR is 3 which leads to a cubic linear function, and the improvement parameter of KRR is 1.0. Before doing the regression task, we scale bone ages from [0, 228] months to uniform float value [0, 1]. At the inference stage, we project their bone age back to the original range, i.e., 0 to 228 months. Before we trained our neural network, we balanced the data by sampling the same number of data with the same bone age. What is more important, not only we transferred the well-trained parameters into our BAA model but also we fine-tuned the parameters in DCNN with SVR or KRR. Cross-validation is employed at the training stage to prevent overfitting and achieve better generalization performance. We set the cross fold as 5.

We use Mean Average Error (MAE), Root Mean Square Error (RMSE), and Concordance Correlation Coefficient (CCC) to evaluate proposed methods. The MAE and RMSE intuitively represent the distance between real and prediction of bone age (lower is better). The CCC has better performance to evaluate the correlation between real bone age and prediction (higher is better) than *R*^2^ score and explained variance score [30]. The experimental results are shown in Table 2.

From Table 2, we observe that our models achieve the best MAE, RMSE, and CCC by using KRR on data transferred via Inception-ResNet-V2. A further crucial observation is that all best measures are acquired in the same setting. Separate regression models for male and female cohorts demonstrate higher accuracy when compared to those trained on a mixed population. With a single regressor, MAEs of the whole dataset, male, and female are 14.83 months, 12.82 months, and 11.93 months, respectively. That suggests that the loss error is about 1 year on average on a single patient.

**Figure 8:**
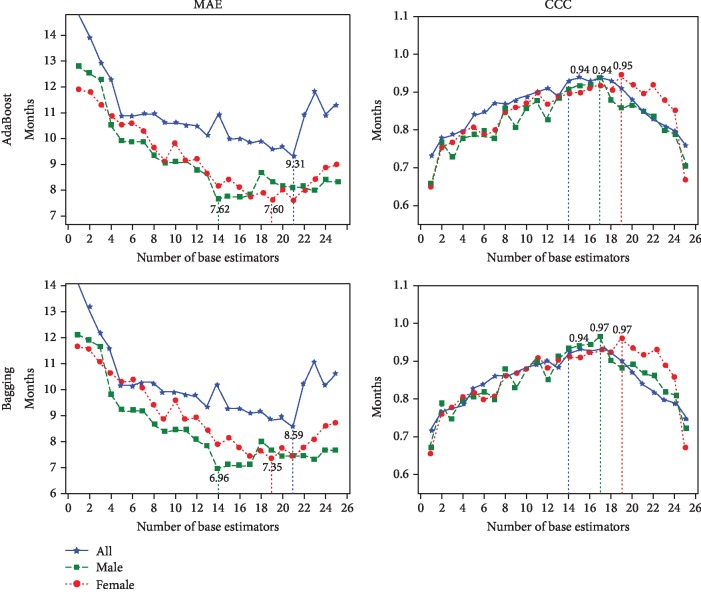
Performance of different ensemble regression methods on data transferred by Inception-ResNet-V2.

To enhance model performance, we employed ensemble learning to lower the regression error further. Ensemble modeling is a powerful way to improve the performance of the low generalized model by combining a diverse set of learners and adjusting data weights in training stage. From Table 1, KRR with Inception-ResNet-V2 achieved the best performance on all of the evaluations, so we employ KRR as a base estimator and ensemble them with the Bagging [31] method and AdaBoost. The performance of ensemble regression is shown in Figure 8.

In Figure 8, the horizontal and vertical axis represents the number of base estimators (KRR) used in ensemble learning and the corresponding evaluations, respectively. From Figure 8, we further enhance the model performance by AdaBoost and Bagging ensemble learning. The final performance of the proposed methods is listed in Table 3. In Table 3, the number in brackets indicates the number of base estimators used in ensemble learning. We lower the error rate about 3 months on each part of dataset compared with a single KRR estimator without Bagging. In our experiment, Bagging outperforms AdaBoost. A further observation is that the best CCC values are enhanced about 0.05 on the male and female dataset. The experimental results suggest that by using ensemble learning, the correlation between real and predicted bone ages is higher than using a single regressor.

Table 4 demonstrates the comparison of the model performance with several existing approaches on BAA tasks. By observing Table 4, it is clear that our proposed model dominates over other methods in part of MAE evaluation.

## 6. Discussion and Conclusions

### 6.1. Discussion

Using our proposed BAA approach, we achieved a MAE of 8.59, 6.96, and 7.35 months on all, male, and female cohorts of the dataset.

Since AL queries unlabeled data and asks Oracle to annotate them, the number of training data is enlarged by the AL strategy and more labeled data available can benefit in training neural networks. More importantly, AL inclines to pick up the most uncertain and informative data for another training epoch so that the active learner learns the most crucial data in training. Essentially, AL boosts the training process so that the trained model can get a better solution.

A further significant investigation is that we proposed a framework of medical image segmentation to relieve human expert annotation burden via deep active learning. Feature vector differences between different members in the committee are taken into consideration. The members can work cooperatively to determine which datum is crucial in the training procedure and then ask oracle to annotate it. In the segmentation stage, benefitting from deep active learning, we only annotated 300 images—about 2.3% of the whole dataset—to make precise hand segmentation.

With the annotated hand X-ray images, our results support the finding by others demonstrating the effectiveness and applicability of transferring deep-learning weights to data from different domains [34]. Transfer learning is important in our framework since the pretrained network is required for successful implementation of clinical decision with a relative small medical image dataset. Furthermore, ensemble learning significantly improves the model performance.

Although the proposed BAA approach achieved a state-of-the-art performance, there are also limitations and some values need to be discussed:

1. Number of members in the committee. Although we found the model performance of segmentation networks are enhanced with the increment of the number of members in the committee, the number of members is hard to determine. Besides, we did not ensemble the well-trained active learners to inference the segmentation results simultaneously
2. The computational complexity of the proposed model. As demonstrated in [36], the total time complexity of all convolutional layers in a deep network is as follows:

(3) $$\begin{array}{c} O\left(\overset{d}{\underset{l=1}{\sum }}n_{l-1}s_{l}^{2}n_{l}m_{l}^{2}\right), \end{array}$$

where *l* is the index of a convolutional layer and *d* is the depth. *n*_l_ is the number of filter in the *l*th layer. *s*_l_ is the spatial size of the filter, and *m*_l_ is the spatial size of the output feature map. From (3), we know the time complexity of DCNN is the combination of each convolutional layer. So the time complexity of the proposed BAA approach is *O*(*n*^6^)

### 6.2. Conclusions

In this paper, we have investigated the application of deep transfer learning on medical images, especially for automated bone age assessment using hand radiographs. We tested several popular off-the-shell deep CNNs trained on the RSNA dataset with 12611 X-ray images. We proved that the transfer learning can cope effectively with bone age assessment task. By using an ensemble technique, our model achieved an MAE of 8.59, 6.96, and 7.35 months on all, male, and female cohorts of the dataset, respectively, comparable to the state-of-the-art performance. Furthermore, we explained which pretrained CNN is better to perform BAA.

In summary, we have created a fully automated, deep learning-based preprocessing pipeline to automatically detect and segment the hand and wrist, standardize the images, and perform BAA with pretrained deep CNNs and high-efficiency regression model. In practice, our system can be easily deployed in the clinical environment on a computer with a single GPU.

## 7. Future Work

The investigation presented in this paper leaves many challenges and issues for future research. We summarize the future work as follows:

1. The proposed BAA framework, which contains image segmentation, feature extraction, and ensemble modules, should be validated on other medical image decision problem
2. To proof the effectiveness of AL framework theoretically. Only if we proof it, could we find how many active learners is enough to form a committee
3. Ensemble the well-trained active learners and generate segmentation result simultaneously by AdaBoost or other ensemble learning algorithms

## Figures and Tables

**Algorithm 1:**
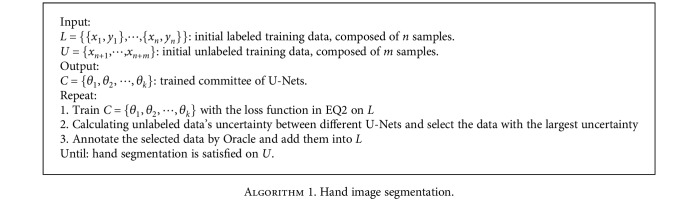
Hand image segmentation.

**Table 1:** Comparison of model performance for hand segmentation.

Strategy	Number of annotated samples	Sensitivity	Specificity	Dice
FSL	300	0.869	0.854	0.869
AL (*k* = 3)	150	0.864	0.845	0.863
AL (*k* = 3)	200	0.902	0.895	0.905
AL (*k* = 3)	300	0.903	0.942	0.939
AL (*k* = 5)	150	0.896	0.909	0.888
AL (*k* = 5)	200	0.904	0.925	0.916
AL (*k* = 5)	300	0.935	0.946	0.931
AL (*k* = 7)	150	0.879	0.902	0.899
AL (*k* = 7)	200	0.932	0.934	0.926
AL (*k* = 7)	300	**0.948**	**0.960**	**0.952**

**Table 2:** Performance of different regression methods on different transferred data.

Model	Sex	Inception-V3			Xception			Inception-ResNet-V2		
		MAE	RMSE	CCC	MAE	RMSE	CCC	MAE	RMSE	CCC
SVR (linear)	All	16.4688	21.1794	0.7139	15.6739	20.3728	0.7029	14.2175	18.0785	0.7143
	Male	12.8732	17.7263	0.5987	11.9983	13.2222	0.6319	11.7378	14.8372	0.6417
	Female	13.2739	17.9381	0.6163	13.6930	14.8271	0.6184	13.0116	17.3823	0.6371

KRR (linear)	All	15.1232	18.2813	0.7004	15.2830	17.7362	0.7793	**13.9381**	**14.8373**	**0.7293**
	Male	13.0293	14.2521	0.6313	12.2321	14.9382	0.6098	**11.9283**	**12.8231**	**0.6563**
	Female	14.7421	19.0855	0.6277	13.3361	17.3211	0.6176	**12.7744**	**11.9321**	**0.6473**

**Table 3:** Performance of different ensemble regression methods.

Ensemble method	Dataset	MAE	CCC
AdaBoost	All	9.31 (21)	0.94 (14)
	Male	7.62 (14)	0.94 (17)
	Female	7.60 (19)	0.95 (19)

Bagging	All	**8.59 (21)**	**0.94 (14)**
	Male	**6.96 (14)**	**0.97 (17)**
	Female	**7.35 (19)**	**0.97 (19)**

**Table 4:** Comparison of approaches in BAA in RSNA dataset.

Method	MAE (m)
Iglovikov et al. [32] without ensemble	8.08
Iglovikov et al. [32] with ensemble	7.52
Wu et al. [33]	7.38
Han et al. [34]	8.40
Tajmir et al. [35]	7.93
Proposed	**7.35**

## Data Availability

The X-ray imaging data used to support the findings of this paper have been deposited in the RSNA repository at doi:10.1148/radiol.2018180736.
